# The antitumor effect of arsenic trioxide on hepatocellular carcinoma is enhanced by andrographolide

**DOI:** 10.18632/oncotarget.18677

**Published:** 2017-06-27

**Authors:** Xuhua Duan, Tengfei Li, Xinwei Han, Jianzhuang Ren, Pengfei Chen, Hao Li, Shaojun Gong

**Affiliations:** ^1^ Department of Interventional Radiology, The First Affiliated Hospital, Zhengzhou University, Zhengzhou, Henan Province, People’s Republic of China

**Keywords:** andrographolide, As_2_O_3_, HepG2 cells, apoptosis, EphB4

## Abstract

High concentrations of arsenic trioxide (As_2_O_3_) are used to treat acute promyelocytic leukemia and solid tumors, with negative side effects to normal cells. Andrographolide is a traditional Chinese medicine that exerts anti-cancer, anti-inflammatory, anti-virus, and anti-diabetic effects. Here, we tested the effects of combined andrographolide with As_2_O_3_ against hepatocellular carcinoma (HCC). We found that by increasing apoptosis, andrographolide synergistically enhanced the anti-tumor effects of As2O3 in HepG2 cells *in vitro* and *in vivo*. Furthermore, results from our microarray assays and experiments with mouse xenografts showed that EphB4 was downregulated by the combination of As_2_O_3_ plus andrographolide. These findings suggest that the combination of andrographolide and As_2_O_3_ could yield therapeutic benefits in the treatment of HCC.

## INTRODUCTION

Hepatocellular carcinoma (HCC), the most common primary malignancy of the liver, causes one million deaths worldwide annually. Approximately 53% of HCC-associated deaths occur in China [1, 2], and the incidence rate has been increasing over the past two decades [3]. In spite of new developments in treatment and clinical prevention strategies, the 5-year survival of HCC patients remains dismally low [4]. The majority of the poor prognoses were associated with drug resistance, cancer recurrence, and metastasis following treatment. Therefore, further research investigating effective therapies for HCC might help prolong the 5-year survival rate.

Arsenic trioxide (As_2_O_3_) has been used in China for more than 2400 years to treat diverse ailments including tuberculosis and gastric ulcers [5]. Recently, As_2_O_3_ has been used to treat cancer [6, 7]. Studies have been focused on the effect of As_2_O_3_ on solid tumors, including osteosarcoma, gastric carcinoma, colorectal carcinoma, and hepatocellular carcinoma, among others [8–11]. However, the high concentrations of As_2_O_3_ required to be effective against solid tumors are toxic to normal cells [12]. Andrographolide, isolated from the traditional medicinal herb *Andrographis paniculate* Nees, exerts several pharmacological effects, such as anti-cancer, anti-inflammatory, anti-virus, and ant-diabetic, among other [13–16]. Recent studies indicated that andrographolide could increase radio-sensitivity, and stimuli-responsive chemotherapy [17, 18]. Therefore, here we propose that andrographolide in combination with As_2_O_3_ could be used as a potential therapeutic agent against HCC. Indeed, andrographolide might act synergistically with As_2_O_3_ to increase the latter’s toxic effects on liver cancer cells at reduced As_2_O_3_ concentrations.

## RESULTS

### Andrographolide synergistically enhanced the anti-tumor effects of As_2_O_3_ in HepG2 cells

**Figure 1:**
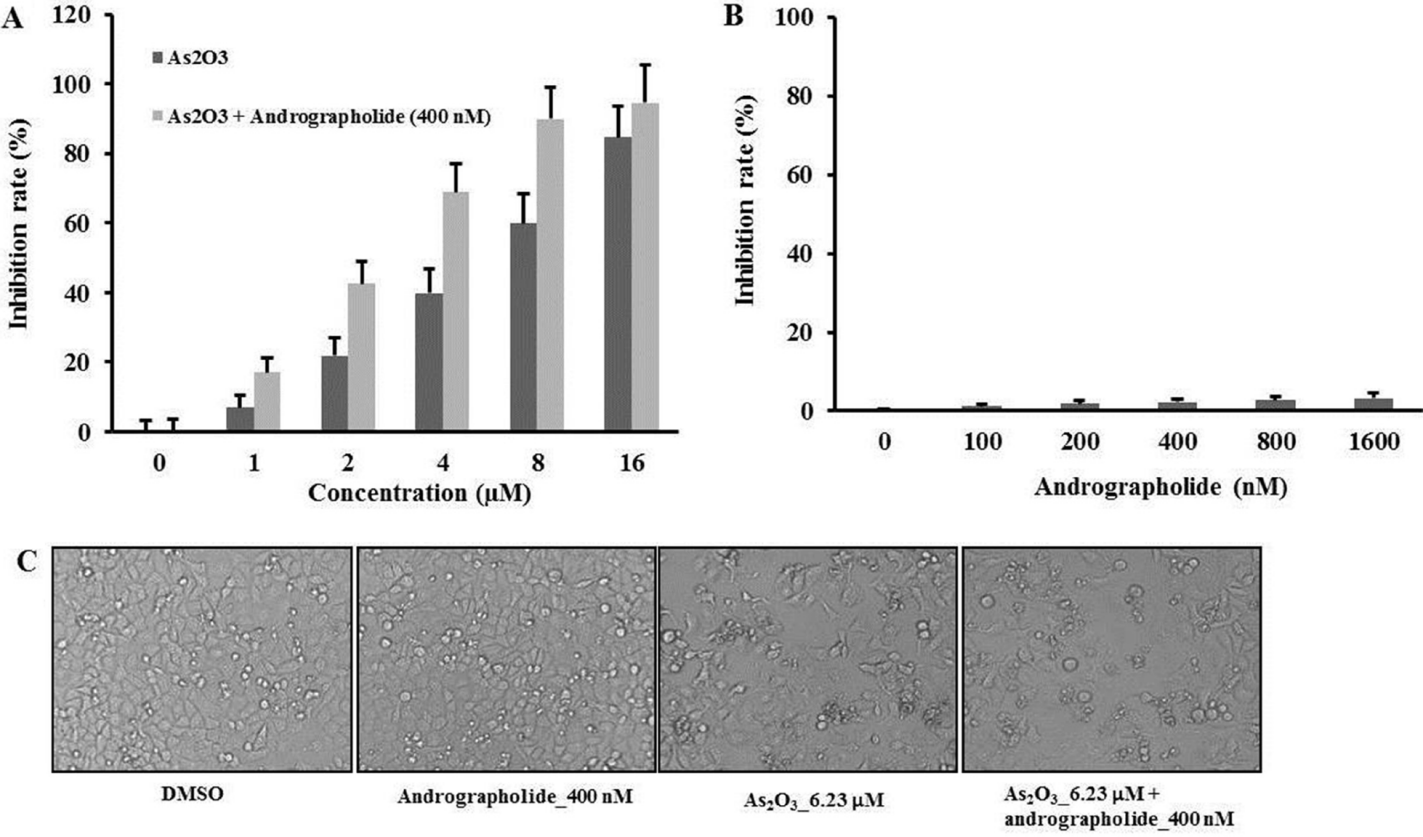
Andrographolide synergistically enhanced the anti-tumor effects of As_2_O_3_ on HepG2 cells (**A**) HepG2 cells were treated with andrographolide from 100 to 1600 nM for 48 h. The growth inhibition rate was measured by MTT assay. (**B**) The cells were treated with As_2_O_3_ or As_2_O_3_ plus andrographolide at indicated time point. (**C**) The cells were treated with indicated treatment for 48 h and morphological changes were visualized using a light microscope at 200 × magnification. Each experiment was repeated independently more than three times.

We used MTT assay to explore the cytotoxicity effect of As_2_O_3_ andrographolide in HepG2 cells. Treatment with As_2_O_3_ alone for 48 h resulted in a dose-dependent inhibition of HepG2 cell growth. Such effect was increased in the presence of 400 nM andrographolide (Figure 1A). Meanwhile, the IC_50_ value of As_2_O_3_ combined with andrographolide was 2.88 μM, which was lower than the value for As_2_O_3_ treatment alone (6.23 μM) (Table 2). We then evaluated the synergistic inhibitory effect of As_2_O_3_ and andrographolide on cell growth by using the combination index (CI) method [19]. The CI value for the combination treatment was 0.68 at IC_50_ of As_2_O_3_ (Table 1). In addition, treatment with andrographolide alone ranging from 0 to 1600 nM for 48 h did not induce cell death (Figure 1B). The morphological changes of HepG2 cells were consistent with cell viability assays (Figure 1C). We used another hepatocellular carcinoma cell line, Huh7, to test the anti-tumor effect of the combination treatment. The results from experiments on Huh7 cells were consistent with those from experiments using HepG2 or SNU449 cells (Supplementary Figure 1A, 1B) These results indicated that andrographolide synergistically enhanced the anti-tumor effects of As_2_O_3_ in Hepatocellular Carcinoma.

**Table 1:** The IC50 and CI values of As_2_O_3_ alone treatment or the combination of As_2_O_3_ with andrographolide in HepG-2 cells

Drugs	IC50 values (µM, 48 h)	CI values at IC50
As_2_O_3_	6.23	−
As_2_O_3_ + 400 nM AND	2.88	0.68

The concentration of As_2_O_3_ ranged from 0 to 16 µM.

**Table 2:** The primer sequences used for real-time polymerase chain reaction

Genes		Primes (5′-3′)
*EphB4*	sense:	CCTTCCTGCGGCTAAACGAC
	antisense:	GTTGACTAGGATGTTGCGAG
*VEGFR1*	sense: antisense:	GAGAGTTCGGCTAGATCAAC CGGATGTAGTTCGCCGAATG
*Ras*	sense: antisense:	TGACGCAGATCAAGTGTGAGA ACTCCATGCACAGGTACGA
*GAPDH*	sense:	GGCATGGACTGTGGTCATGAG
	antisense:	TGCACCACCAACTGCTTAGC

### Andrographolide’s synergistic enhancement of As_2_O_3’s_ anti-tumor effects was independent of autophagy

**Figure 2:**
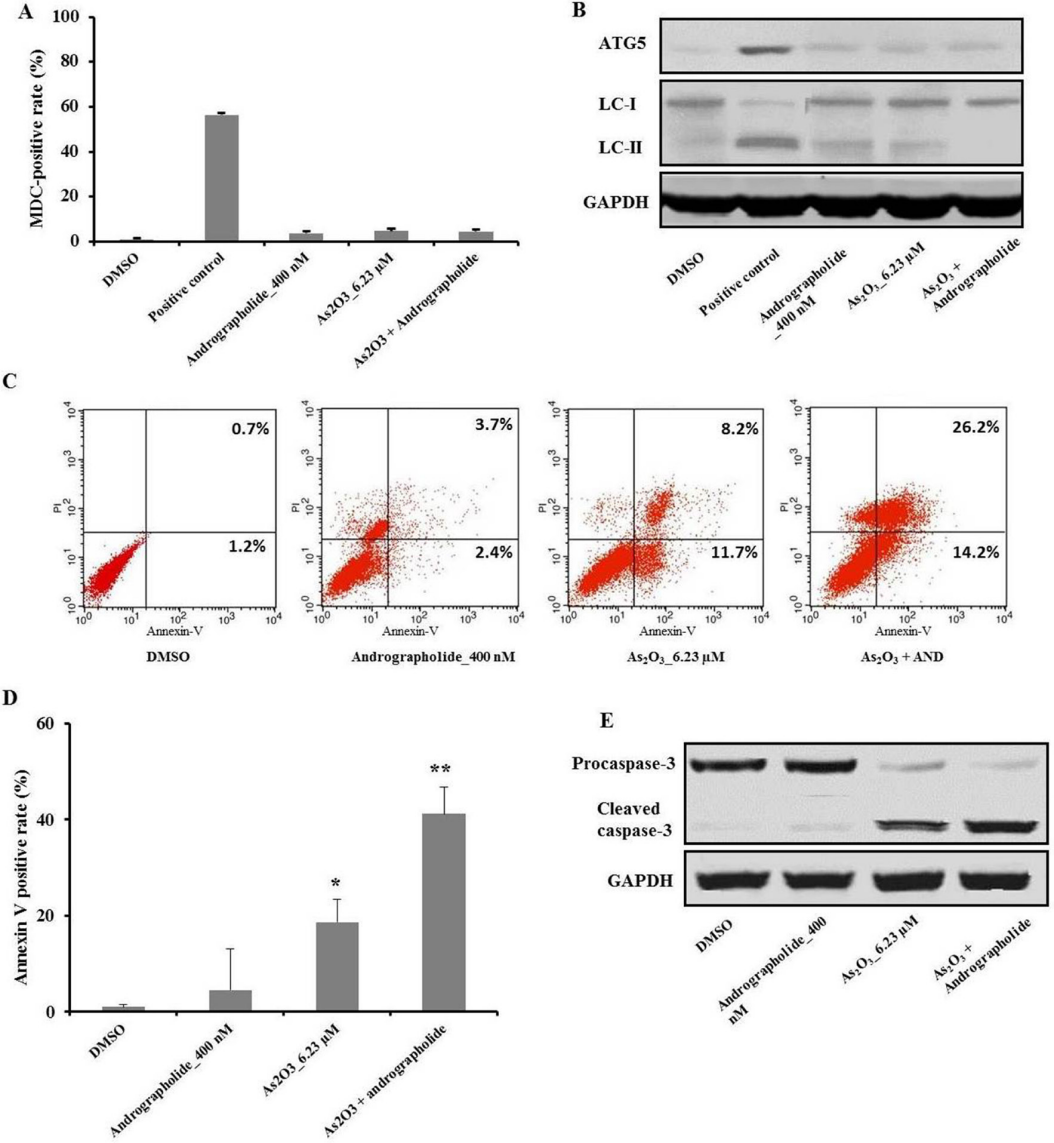
Andrographolide increased As_2_O_3_-induced apoptosis in HepG2 cells, which was independent of autophagy (**A**) HepG2 cells were treated with the indicated treatments for 48 h, and then incubated with MDC at 0.05 mM for 10 min at 37°C. After washing twice with PBS, the cells were immediately analyzed by flow cytometry. (**B**) The levels of Atg-5 and LC-3 were analyzed using western blot. (**C**–**D**) The cells were treated with the indicated treatments and, after an additional incubation for 48 h, the cells were stained with Annexin V and PI. Apoptotic cells were analyzed using flow cytometry. The fraction of viable cells: Annexin V^−^, PI^−^; lower left quadrant. Necrotic cells: Annexin V^−^, PI^−^; upper left; of early apoptotic cells: Annexin V^−^, PI^−^; lower right; of late apoptotic cells: Annexin V^−^, PI^−^. (**E**) The level of cleaved caspase-3 was analyzed by western blot. Each experiment was repeated independently more than three times. *P < 0.05, **P < 0.01 by two-tailed *t*-test.

We hypothesized that the combination of andrographolide plus As_2_O_3_ induces autophagy on HepG2 cells. To test this, we performed MDC staining, which can reveal autophagic vacuoles in flow cytometry experiments. MDC staining in the presence of andrographolide and/or As_2_O_3_ was negative compared with the positive control (Figure 2A). The conversion of LC3-I into LC3-II by cleavage and the expression of autophagy-related gene 5 (Atg 5) are hallmarks of autophagy activation. Therefore, we measured the levels of LC3-II and ATG-5 and found that andrographolide and/or As_2_O_3_ did not induce autophagy (Figure 2B).

### Andrographolide increased As_2_O_3_-induced apoptosis in HepG2 cells

To investigate the mode of cell death induced by the combination treatment of andrographolide with As_2_O_3_, we labeled cells with Annexin V and PI using flow cytometry [20]. Compared with the As_2_O_3_ treatment group, the combination therapy had a higher apoptotic rate in HepG2 (Figure 2C, 2D). On the other hand, treatment with andrographolide alone did not induce apoptosis, in agreement with cell viability assays.

Caspases are a family of cysteinyl aspartate-specific proteases, which are involved in cell apoptosis, with caspase-3 acting as the executioner caspase [21]. Western blot analysis showed that the combination treatment of As_2_O_3_ plus andrographolide yielded higher levels of cleaved caspase-3 compared with As_2_O_3_ treatment alone. On the other hand, andrographolide alone did not affect the level of cleaved caspase-3 (Figure 2E). Taken together, these results indicated that andrographolide synergistically increased As_2_O_3_-induced apoptosis in HepG2 cells in a caspase-dependent manner.

### Andrographolide synergistically enhanced the anti-proliferative effect of As_2_O_3_ in HepG2 cells and downregulated EphB4

**Figure 3:**
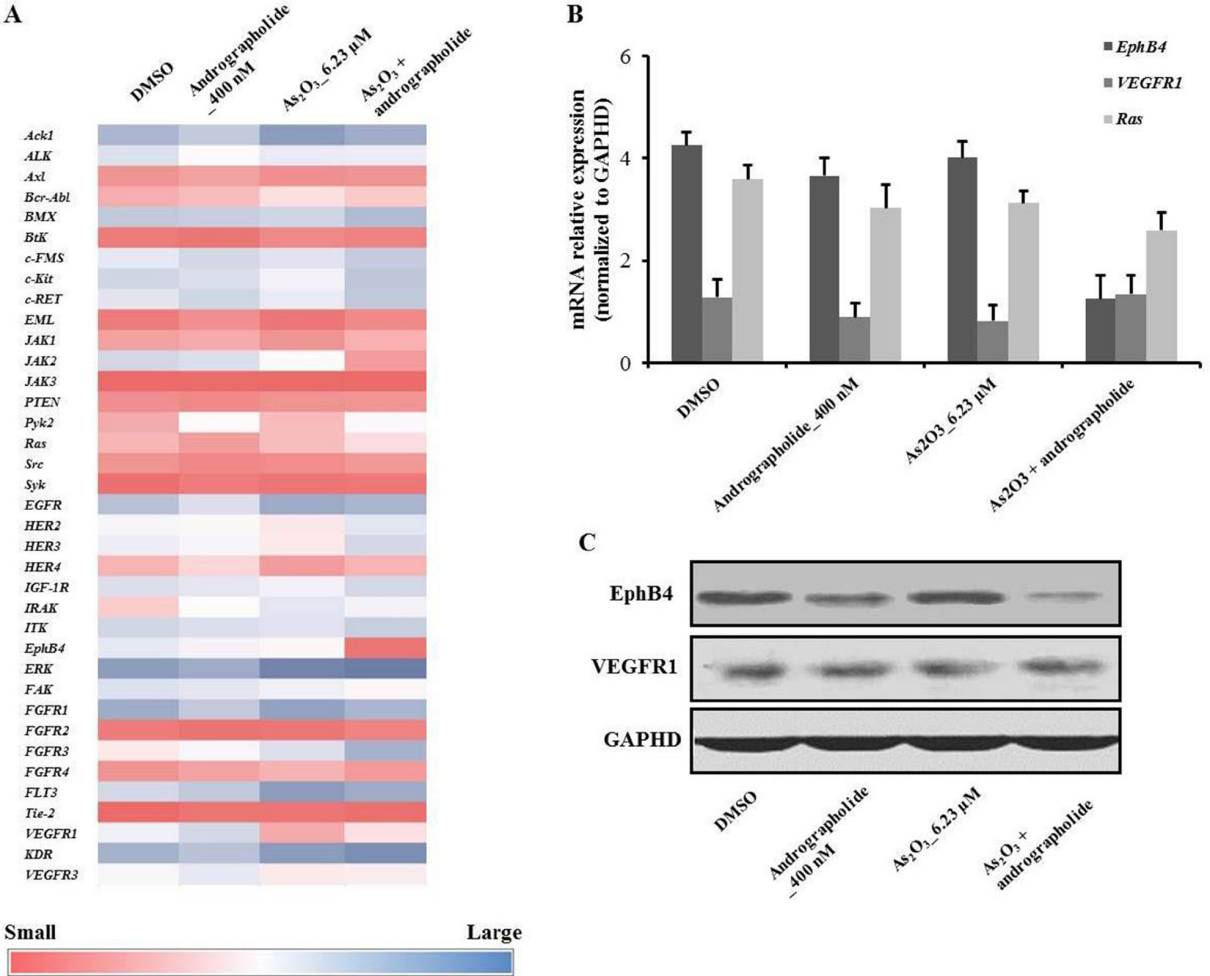
Andrographolide synergistically enhanced the anti-proliferative effects of As_2_O_3_ on HepG2 cells via downregulation of EphB4 (**A**) Heat map showing the effect of andrographolide plus As_2_O_3_ on the expression of HepG2 genes involved in andrographolide’s synergistic anti-cancer activity. (**B**) Quantitative RT-PCR was used to evaluate the levels of *EphB4*, *Ras* and *VEGFR1* in HepG2 cells. (**C**) The expression of EphB4 and VEGFR1 were analyzed by western blot. Each experiment was repeated independently more than three times.

We performed microarray analysis to identify differences in the expression of 37 genes under various treatments (Figure 3A). Among the examined genes, *Ras*, *VEGFR*, and *EphB4* showed differential expression (Figure 3A). RT-PCR analysis confirmed that the expression of *EphB4* was decreased in the presence of andrographolide plus As_2_O_3_ in HepG2 cells (Figure 3B). On the other hand, the levels of oncogenes *Ras* and *VEGFR1* were unchanged. In addition, we also used western blot to measure the levels of EphB4 and VEGFR1 in HepG2 cells under the indicated treatments. The results showed that the level of EphB4 was decreased under the combination treatment, indicating that EphB4 was affected by the synergy between andrographolide and As_2_O_3_ (Figure 3C).

### Andrographolide enhanced the anti-tumor effect of As_2_O_3_ in HepG2 xenografts and the combination treatment inhibited the EphB4 pathway *in vivo*

**Figure 4:**
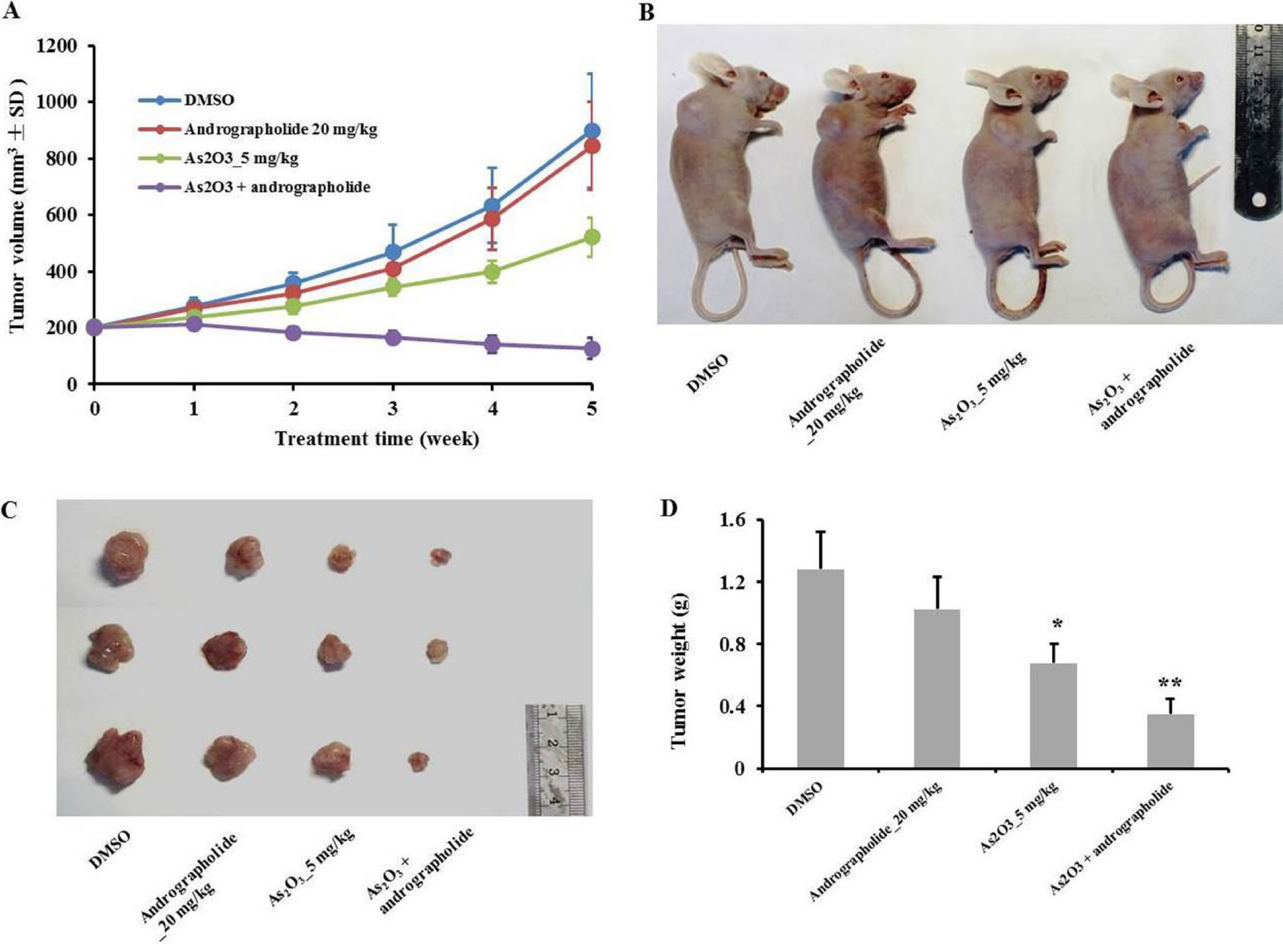
Andrographolide synergy enhanced the anti-tumor effect of As_2_O_3_ *in vivo* (**A**) Tumor volumes of HepG2 xenografts were monitored weekly in each group. (**B**) Tumor bearing mice were sacrificed 5 weeks after treatment. (**C**) Tumors were resected from nude mice and weighted 5 weeks after treatment. (**D**) Graphs showing the average weights of harvested xenograft tumors (*n* = 6). *P < 0.05, **P < 0.01 by two-tailed *t*-test.

We next tested the effect of the combination treatment *in vivo* using HepG2 xenografts, choosing doses of andrographolide as previously described [13]. We also performed a pilot study to determine adequate doses for the combination treatment (Supplementary Figure 2). Tumor growth was inhibited after i.p. injection of andrographolide plus As_2_O_3_ for five weeks, compared with andrographolide or As_2_O_3_ monotherapy (Figure 4A). As_2_O_3_ monotherapy also inhibited the growth of tumors at a dose of 5 mg/kg. At the end of the study, the mice were sacrificed, and the tumors were weighed. The combination treatment dramatically decreased tumor weights comparing with the other treatments (Figure 4C, 4D). In conclusion, andrographolide enhanced the anti-tumor effect of As_2_O_3_ in HepG2 xenografts.

**Figure 5:**
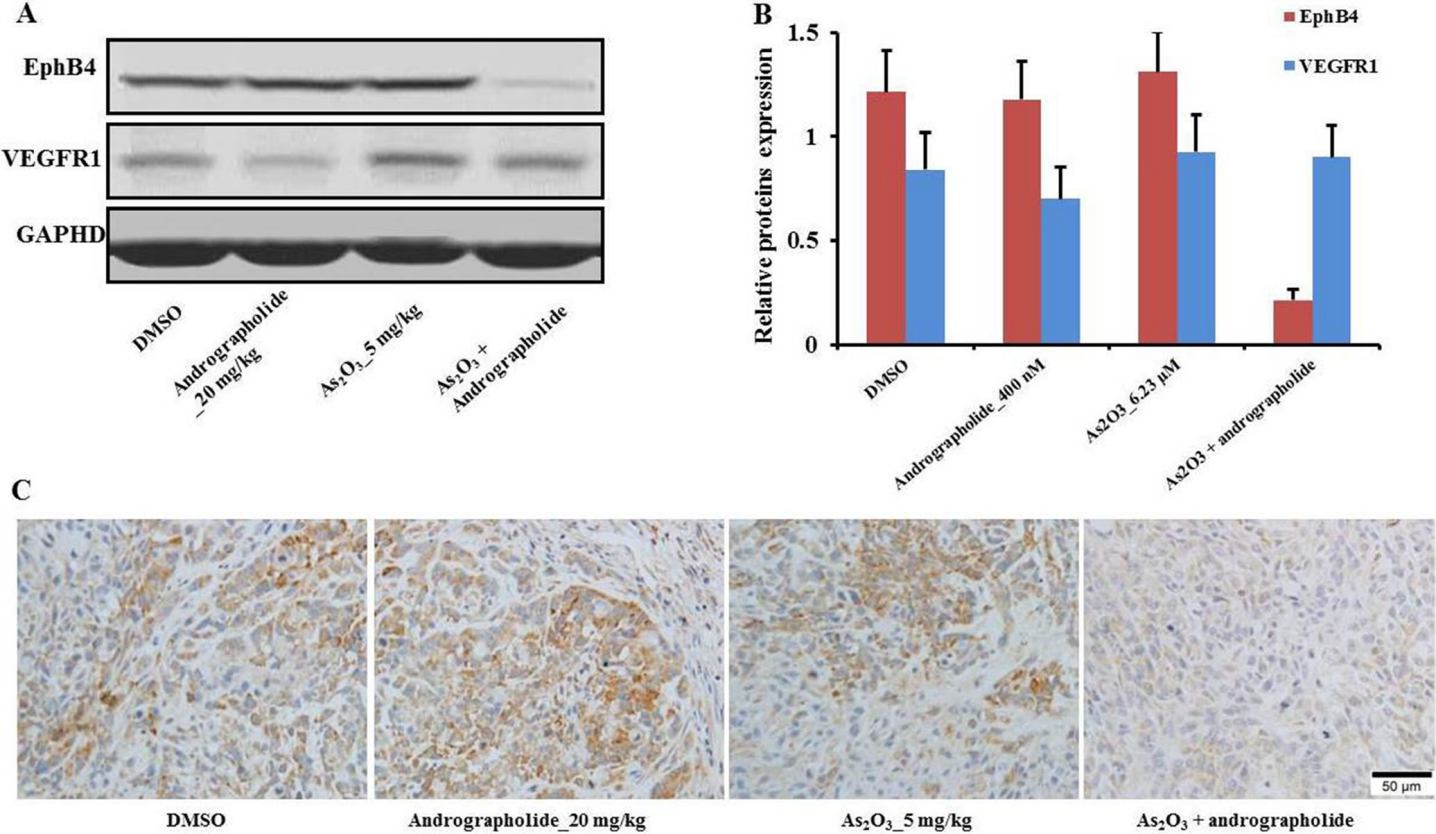
EphB4 downregulation enhanced andrographolide’s promotion of As_2_O_3_ anti-tumor effects *in vivo* (**A**) The levels of EphB4 and VEGFR1 were analyzed by western blot assay. (**B**) Quantitative results by western blot. (**C**) Immunohistochemical validation of EphB4 in representative HCC and control tissue sections stained with anti-EphB4 antibody. Each experiment was independently repeated more than three times.

Next, we found that the expression of EphB4 in tumor tissues was also decreased in the combination treatment group compared with the other treatment groups (Figure 5A, 5B). These results were consistent with *in vitro* data from western blot and RT-PCR experiments. Moreover, immunohistochemical analysis of tumors confirmed that EphB4 levels were decreased in the combination treatment group compared to the other treatment groups (Figure 5C). These results indicated that EphB4 downregulation correlated with the inhibitory effect of the combination treatment in HepG2 cells *in vivo*.

### Andrographolide increased As_2_O_3_-induced apoptosis *in vivo*

**Figure 6:**
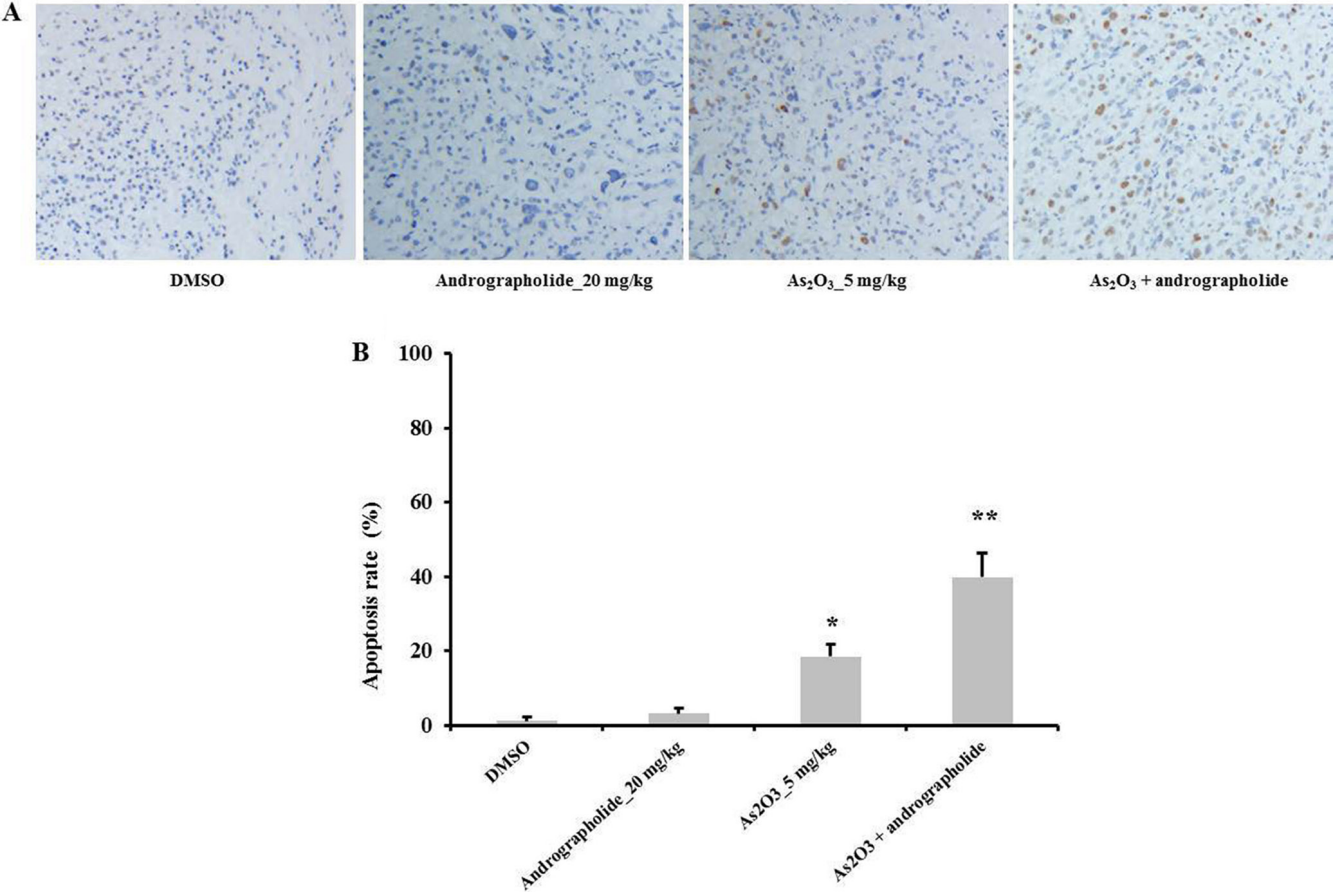
Andrographolide enhanced As_2_O_3_-induce apoptosis *in vivo* (**A**–**B**) TUNEL assay was performed to determine the level of apoptosis in HepG2 tumor tissues (*n* = 3). *P < 0.05, **P < 0.01 by two-tailed *t*-test.

We used TUNEL assay to measure the apoptotic effect of the combination treatment *in vivo*. Our results showed that the combination treatment induced a higher apoptosis rate *in vivo* compared with andrographolide or As_2_O_3_ alone. Indeed, andrographolide alone did not induce apoptosis at all (Figure 6A, 6B). These data showed that andrographolide increased As_2_O_3_-induced apoptosis *in vivo*. These results were consistent with our *in vitro* experiments.

## DISCUSSION

Hepatocellular carcinoma is the leading cause of death in Asia, the second cause of cancer death in China, and its incidence is increasing in western countries [1, 22]. As_2_O_3_ is one of the major cytotoxic agents to treat acute promyelocytic leukemia (APL) and other leukemic cancer cells [5]. At therapeutic doses, As_2_O_3_ causes adverse side effects such as sever bone marrow depression, secondary malignancies, and cellular toxicity [23, 24]. Indeed, As_2_O_3_ can exert anti-tumor effects on solid tumor cells such as HepG2 cells, but the effective concentration needs to be higher than 10 μM [25]. HepG2 cells are more resistant to As_2_O_3_ than APL cells (effective dosage < 10 μM); therefore, drug resistance and chemotoxicity limit the therapeutic efficacy of As_2_O_3_ in treating solid HepG2 tumors [26–28]. In the present study, we discovered that andrographolide synergized with As_2_O_3_ to increase the latter’s toxicity, leading to increased apoptosis in HepG2 cells *in vitro* and *in vivo*. Andrographolide was also able to enhance As_2_O_3_-mediated cell growth inhibition. The results were supported by the activation of cleaved caspase-3. Furthermore, we uncovered the underlying mechanism of apoptotic potentiation, which involves EphB4 downregulation upon treatment andrographolide plus As_2_O_3_ treatment.

As_2_O_3_ induces apoptosis in HCC [29, 30] and andrographolide acts in synergy with As_2_O_3_ to induce cell death by abrogation of As_2_O_3_-induced G2/M arrest [28]. In the present study, by activating cleaved caspase-3, andrographolide promoted As_2_O_3_’s induction of apoptosis. Furthermore, our MDC staining experiments indicated that autophagy was not elicited by the combination treatment in HepG2 cells. In agreement with this, gene expression tests for LC3 and Atg-5 were negative. Additionally, results from *in vivo* experiments on xenografts further substantiated our findings. Our results demonstrated that andrographolide enhanced the anti-tumor effects of of As_2_O_3_ in HepG2 cells.

EphB4 is a member of the receptor tyrosine kinase (RTK) family, which promotes tumor tissue development, oncogenesis and progression [31–33]. We used microarray assays to study the mechanism underlying the synergism between As_2_O_3_ and andrographolide in HCC. Our data revealed that the expression of EphB4 in the combination treatment group was much lower than that in the groups treated with As_2_O_3_ or andrographolide alone *in vitro*. Results from RT-PCR and western blot experiments *in vitro* and *in vivo* were consistent with this finding. Additionally, our immunological assays also indicated that EphB4 was downregulated by the combination treatment. However, conflicting results have been reported for EphB4 in different tumor cells. A previous study reported that EphB4 is an important indicator of poorly differentiated hepatocarcinoma, with EphB4 expression correlating with angiogenesis and tumor progression [34]. Furthermore, overexpression of EphB4 has been found in several tumor types including ovarian and prostate cancer, among others [35]. Lastly, in our experimental systems the expression of oncogenes *Ras* and *VEGFR1* was unchanged. Therefore, further research is needed to uncover the mechanisms underlying EphB4 downregulation and its involvement in HCC tumor cell apoptosis.

In summary, we demonstrated that the andrographolide synergistically enhances As_2_O_3_’s antitumor activity in HCC cells *in vitro* and *in vivo*. Specifically, andrographolide enhanced As_2_O_3_-induced apoptosis in a caspase-3 dependent manner via downregulation of EphB4. Thus, our data suggest that lower doses of As_2_O_3_ in combination with andrographolide could be used as chemotherapy for HCC with the potential to minimize the toxic side effects from As_2_O_3_ treatment alone.

## MATERIALS AND METHODS

### Reagents

Andrographolide (purity > 98%) was purchased from the National Institutes for Food and Drug Control, China. Arsenic trioxide (As_2_O_3_), trypsin, phosphate buffer saline (PBS), ethylene diamine tetraacetic acid (EDTA), 3-(4,5-dimethyl-2-thiazolyl)-2,5-diphenyl-2-H-tetrazolium bromide (MTT), monodansylcadaverine (MDC), propidium iodide (PI), Annexin V, and rapamycin were purchased from Sigma Chemical Corp. (St. Louis, MO, USA). Primary antibodies against caspase-3, LC3, Atg-5, EphB4 and VEGFR1 were purchased from Santa Cruz Biotechnology (Santa Cruz, CA, USA). All the other chemical reagents in the study were of analytical reagent grade.

### Cell culture

The human hepatoma cell lines HepG2, Huh7 and SNU-449 were obtained from the American Type Culture Collection (ATCC, Manassas, VA, USA) in 2016. The cells were cultured in RPMI1640 supplemented with 100 μg/mL streptomycin, 100/mL penicillin and 10% fetal bovine serum at the atmosphere of 37°C with 5% CO_2._

### Cell viability assay

HepG2, Huh7 or SNU-449 cells were seeded into 96-well plates (Corning, NY, USA) at a density of 1 ×10^4^ cells/well. The culture medium was replaced with fresh medium after 48 h incubation and the cells were subjected to appropriate treatments. Then the cell viability was measured by MTT assay. Cells were cultured with 5 mg/mL MTT solution for 3 h at 37°C. Then the culture medium was discarded and formazan produced was dissolved in dimethyl sulfoxide (DMSO) by shaking for 15 min. Absorbance at 492 nm was measured with a microplate reader (Thermo Fisher Scientific). Cell inhibitory ratio was calculated as follow formula:

Cell growth inhibitory ratio(%)=100×(A490,control−A490,sample)(A490,control−A490,blank) 1

### Observation of cell morphological changes

HepG2 cells were seeded into 6-well plates at a density of 3 × 10^5^ cell/well (Corning, NY, USA). Then, the cells were treated with indicated concentrations of andrographolide or/and As_2_O_3_ for 48 h. The cellular morphologies were observed and captured by phase contrast microscope (Olympus America Inc., Center Valley, USA).

### Analysis of drug synergism

Synergism analysis was performed following the method described previously [36]. Data from cell viability assay were employed to perform this statistical analysis. The combination-index (CI) was used to evaluate the influence of each of the two drugs independently, as well as both drugs simultaneously, calculated as follows:

$$\text{Combination Index}\left(\mathrm{CI}\right){=D}_{1}/{\left(\mathrm{DX}\right)}_{1}+D_{2}/{\left(\mathrm{DX}\right)}_{2}$$

D1 and D2 represent the concentration of combination treatment of drug 1 and drug 2 achieving x% of the total drug effect, whereas (D_X_)_1_ and (D_X_)_2_ are the concentrations of drug 1 or drug 2, respectively, to achieve the same efficacy. The combination was synergistic when CI < 1, and antagonistic when CI > 1, and additive when CI = 1.

### Cell apoptosis assays

PI, plasma membrane-impermeant fluorochrome, was used as an exclusion dye. Annexin V was used to detect phosphatidylserine (PS) exposure on the outer leaf of the plasma membrane. Apoptosis was quantitatively investigated by analysis of PI and Annexin V labelling using flow cytometry as previously reported [37]. Briefly, cells were collected after different treatments and incubation at 37°C for 48 h, washed with cold PBS twice and centrifuged at 1200 rpm for 5 min. Then, the cells were suspended with mixed buffer supplemented with 5 μL Annexin V and 5 μL and PI (Pierce Biotechnology, Rockford, IL, USA). After incubation at 4°C for 30 min in the dark, the cells were analyzed by flow cytometry.

### MDC staining assay

MDC is an effective fluorescent dye used to detect autophagic vacuoles, which are part of autophagosomes [38]. HepG2 cells were seeded into 6-well plates and treated with AND or/and As_2_O_3_ treatments, and 100 nM rapamycin was used as a positive control. After 48 h incubation, the cells were trypsinized, resuspended in DMEM medium, and centrifuged at 400 × *g* for 10 min. Then, the cells were washed with 1 × cold PBS twice and resuspended, added into MDC solution (20×), and incubated at 37°C for 40 min. The autophagosomes were analyzed by a flow cytometry (BD Biosciences, Franklin Lake, NJ, USA). Rapamycin (200 nM) was used as a positive control.

### Western blotting assay

In order to collect proteins from cultured cells and tumors, the samples were washed with PBS and extracted using cell lysis buffer (GeneHunter, Basgvukke, TN, USA). After 20 min standing, the lysates were centrifuged at 12 000 × *g* for 10 min at 4°C. The protein concentration was measured by Bio-Rad protein assay (Bio-Rad, Hercules, CA, USA). Equal amounts of proteins were subjected to 10% SDS-PAGE gel for electrophoresis for 2 h, then transferred to Millipore Immobilon-P Transfer Membrane (Millipore Corporation, Billerica, MA, USA) at 100 mA for 2 h. Western blot analysis was performed with primary antibodies against ATG-5, LC3-I, LC3-II, GAPDH, caspase-3, EphB4, and VEGFR-1, and incubated at 4°C overnight. We washed the membranes and probed them with appropriate secondary antibodies for 2 h at room temperature. We measured protein levels using electrochemiluminescence (ECL) (Thermo Fisher Scientific, Rockford, IL, USA).

### Microarrays and gene expression analysis

HepG2 cells were treated with 400 mM AND, 6.23 μM As_2_O_3,_ combination treatment, or vehicle (0.1% DMSO) for 48 h. After treatment, the cells were washed with PBS twice and suspended in 1.0 mL Trizol reagent (Life Technologies, Inc., Carlsbad, CA, USA). Then, microarray analysis was performed using Kangchen Bio-Tech (Shanghai, China) according to Agilent Whole Human genome Oligo microarray protocol. The microarray data were accessible via Gene Expression Omnibus.

### Quantitative real-time PCR analysis

To validate the effect of combination treatment of andrographolide with As_2_O_3_ on the levels of EphB4 *in vivo* and *in vitro*, we performed qRT-PCR analysis. The mice were sacrificed and tumors were excised and minced into small pieces and then homogenized. In addition, HepG2 cells were treated with the indicated treatments for 48 h. Then, total RNA was isolated from tumor tissues and HepG2 cells using Trizol reagent (Life Technologies, Inc., Carlsbad, CA, USA) and treated with DNase I according to the manufacturer’s instructions. We used Nano assay kit (Nanodrop Technologies, Wilmington, DE, USA) to test for RNA integrity. Amplification of cDNA was performed by real time PCR using the appropriate prime pairs: EphB4 or VEGFR-1. All primers were purchased from Sigma Aldrich (St. Louis, MO, USA) and are listed in Table 2. Quantitative PCR was performed using the CFX Connect Real-Time PCR Detection System (Bio-Rad Laboratories, Hercules, CA, USA).

### Animal experiments

Thirty-two male BALB/c six-week-old nude mice were purchased from Vital River Laboratory Animal Technology Co., Ltd (Beijing, China). The mice were housed in a pathogen-free environment and all animals received human care according to Chinese legal requirements. HepG2 cells (3 × 10^6^ cells) were injected subcutaneously into the dorsal flanks of mice. Tumor volumes were calculated as V (mm^3^) = 0.5 × L × W× H. When the tumor volume reached 200 mm^3^, the tumor-bearing mice were randomly divided into four groups. The mice were administered intraperitoneal injections (i.p.) of vehicle control (0.1% DMSO), andrographolide (20 mg/kg), As_2_O_3_ (5 mg/kg), or the As_2_O_3_ with andrographolide (“combination treatment”) for five weeks. The body weights and tumor volumes of xenografts were monitored weekly. At the end of the study, the mice were euthanized, and the tumors were harvested and weighed. All animal experiments were performed in accordance with protocols approved by The United Kingdom Coordinating Committee on Cancer Prevention Research’s Guidelines for the Welfare of Animals in Experimental Neoplasia.

### TUNEL assay

We performed TUNEL staining using a commercial kit (Roche Molecular Biochemicals, Indianapolis, IN) according to manufacturer’s protocol to determine the mode of cell death *in vivo*. In brief, tissue sections were fixed with 1% paraformaldehyde for 15 min and incubated with a cold solution of ethanol:acetic acid (2:1) for an additional 5 min. The sections were subsequently rinsed twice using PBS, immersed in 0.5% Triton for 15 min at 4°C, then washed twice and digested with endogenous peroxidase with 3% H_2_O_2_ for 20 min. After washing twice with PBS, the slides were incubated with TdT enzyme at 37°C for 90 min and 3% hydrogen peroxide for 5 min to block endogenous peroxidase activity, and then treated with peroxidase conjugated antibody for 10 min at room temperature. Finally, the sections were examined by light microscopy.

### Immunohistochemical staining assay

Immunohistochemical staining was performed as previously described [39] to confirm the expression of EphB4 in HepG2 *in vivo*. Briefly, the sections were deparaffinized in xylene and then rehydrated in graded ethanol series prior to antigen retrieval for 20 min. Later on, the sections were washed with PBS and blocked with 5% bovine serum albumin (BSA) for 1 h at room temperature and then washed with PBS. Sections were then incubated overnight at 4°C with primary antibody polyclonal rabbit anti-EphB4 (1:500). The cells were washed again with PBS buffer and then incubated with secondary antibody for 1 h at room temperature. The staining was developed for 5 min using DAB chromogen (Dako, Glostrup, Denmark) and then each section was visualized with a light microscope.

### Statistical analysis

All statistical data are presented as mean ± SD. Student’s *t*-test was applied to determine the statistical significance between control and experimental data. * and * * represent *p* < 0.05 and *p* < 0.01, respectively, for the difference between the experimental data and the control or negative group.

## SUPPLEMENTARY MATERIALS FIGURES


