# Supplementary material for: The antitumor effect of arsenic trioxide on hepatocellular carcinoma is enhanced by andrographolide
Source: Oncotarget. 2017 Jun 27;8(53):90905–15. doi: 10.18632/oncotarget.18677 (PMC5710893; doi:10.18632/oncotarget.18677)
Supplement: Supplementary file 1 [file oncotarget-08-90905-s001.pdf]

The antitumor effect of arsenic trioxide on hepatocellular carcinoma is enhanced by andrographolide

Supplementary Materials

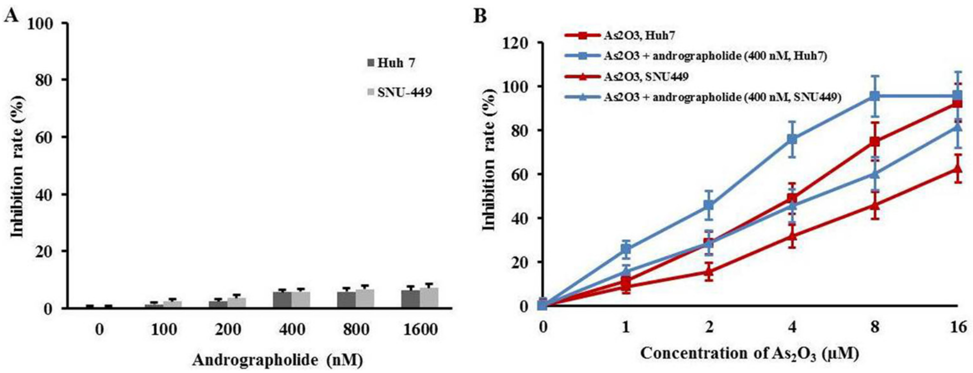

Supplementary Figure 1: Andrographolide synergistically enhanced the anti-tumor effects of As_2O_3 in Huh7 cells. (**A**) Huh7 cells were treated with different doses of andrographolide ranging from 100 to 1600 nM for 48 h. The growth inhibition rate was measured by MTT assay. (**B**) The cells were treated with As_2O_3 or As_2O_3 plus andrographolide at the indicated time point. Each experiment was repeated independently more than three times.

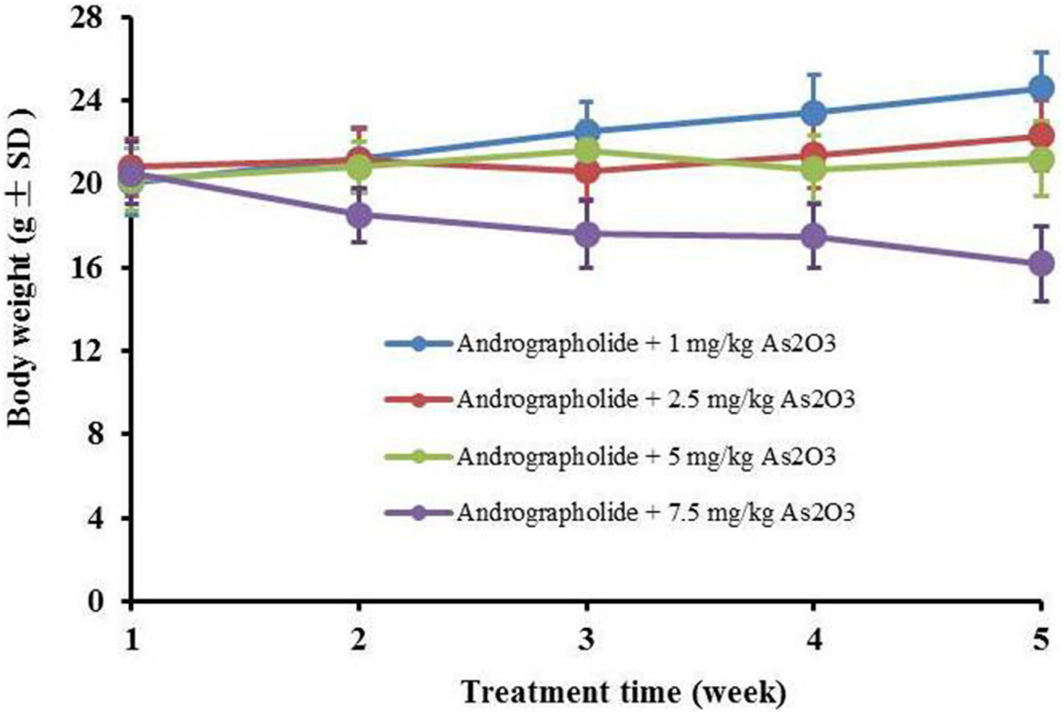

Supplementary Figure 2: Dosage selection for the combination study in HepG2 xenografts *in vivo*. Tumor bearing mice were treated with different combination treatments and their body weights were measured on the indicated days (n = 3).
